# What Has Changed over Years on Complementary Feeding in Italy: An Update

**DOI:** 10.3390/nu15051280

**Published:** 2023-03-04

**Authors:** Marco Congiu, Valeria Cimador, Irene Bettini, Teresa Rongai, Flavio Labriola, Francesca Sbravati, Caterina Marcato, Patrizia Alvisi

**Affiliations:** 1 Residency School of Pediatrics, University of Bologna, 40138 Bologna, Italy; 2 Primary Care Pediatrician, 00183 Rome, Italy; 3 Pediatric Gastroenterology Unit, Maggiore Hospital, 40133 Bologna, Italy

**Keywords:** complementary feeding, baby-led weaning, Italy, baby foods, primary care pediatrician

## Abstract

Current practice regarding complementary feeding (CF) is influenced by socio-cultural background. Our group already investigated the Italian approach to CF in the years 2015–2017. Our aim was to update those data by finding out: if the habits have changed nationwide, how the trends changed in each area, and if the differences between regions still exist. We devised and submitted to Italian primary care paediatricians (PCP) a questionnaire consisting of four items regarding the suggestions they gave to families about CF and compared the results to the ones from our previous survey. We collected 595 responses. Traditional weaning was the most recommended method, with a significant reduction compared to the period of 2015–2017 (41% vs. 60%); conversely, the proportion of PCP endorsing baby-led weaning (BLW) or traditional spoon-feeding with adult food tastings has increased, while the endorsement of commercial baby foods dropped. BLW is still more popular in the North and Centre compared to the South (24.9%, 22.3%, and 16.7%, respectively). The age to start CF and the habit of giving written information have not changed over time. Our results highlighted that Italian paediatricians encourage BLW and traditional CF with adult tastings more than in the past, at the expense of traditional spoon-feeding.

## 1. Introduction

The definition of complementary feeding (CF) given by the World Health Organization (WHO, Geneva, Switzerland) in 2002 is “the process starting when breast milk alone is no longer sufficient to meet the nutritional requirements of infants” so that “other foods and liquids are needed, along with breast milk” [1]. CF represents an essential milestone for a child’s growth but also a delicate and worrisome phase for parents. It is important not only for the child’s thriving but also for his or her neurological development. CF plays a key role in developing a taste for different foods [2] and represents an irreplaceable occasion to educate the taste for the following years, as the child wants to experience foods other than milk: a recent study pointed out that providing vegetables as first foods may be an effective strategy for improving vegetable consumption in infancy [3].

Whereas it is widely accepted that breast milk is the most adequate food in children up to approximately six months of age, there is much debate on the modalities to implement CF. These are indeed heavily influenced by socio-cultural context, paediatricians’ preferences and the families’ inclinations and are, therefore, various and diverse. We report the definitions of the main modalities to implement CF; however, these are not universal and are prone to interpretation. Therefore, they may assume different characteristics in different parts of the world:

- “Traditional spoon-feeding”: the parent spoon-feeds the child, which remains mostly passive in the process; the diet comprises homemade liquid or semi-liquid foods or commercial baby foods. Italian families usually feed their children, as a first food, a meal made of vegetable broth with semolina or rice flour, meat, olive oil and parmesan cheese.
- “Traditional spoon-feeding with adult food tastings”: while following a traditional CF style, in this case, parents occasionally mash and mince the food they are eating and feed it to their child.
- Baby-Led Weaning (BLW): parents are responsible for what, when and where the infant is fed by providing chunks of food but allowing the infant to bring the food to the mouth and deciding on how much food to consume [4].
  ○ “Self-weaning”: an on-demand approach that can be considered the Italian take on BLW; the food offered to the infant is partly the same as that of the parents; the main difference is that here the food is minced, mashed and spoon-fed.

Among these different modalities, BLW has quickly gained popularity in the past few years in Western countries [4]. In a recent Italian study, the frequency of a BLW approach was positively related to breastfeeding, later exposure to complementary foods, earlier exposure to both finger and family foods, and higher interest in family food and shared family meals [5].

Due to its pivotal role in the infant’s present and future well-being, there has been much interest recently in investigating the most adequate way to implement CF. The most recent recommendation update was recently produced by the Italian Society for Preventive and Social Pediatrics (SIPPS), the Italian Society for Developmental Origins of Health and Disease (SIDOHaD), the Italian Federation of Pediatricians (FIMP), and the Italian Society of Pediatric Nutrition (SINUPE) Joint Working Group: their indications are strictly evidence-based and cover several aspects of CF [6]. It is apparent how old beliefs are being brought into question, e.g., no longer is it recommended to postpone or bring forward the introduction of allergenic foods to reduce the risk of allergy [6,7].

The Italian National Healthcare System provides all families with a primary care paediatrician (PCP) who has an established role in caring for the pediatric population during infancy, childhood, and adolescence, with particular attention to their growth and overall health. This includes, among other things, counselling the families about nutrition and the proper way to implement weaning. In order to better understand the current Italian situation regarding CF, in our previous study, we described CF practices in Italy and the differences existing among different geographical areas, considering the timespan from January 2015 to December 2017 [8]. Since many aspects of CF are quickly changing, we submitted the same questionnaire to primary care paediatricians during the year 2022. Therefore, the objective of this study is to compare how CF has evolved over this period, investigating: (1) if the habits have changed on a national basis, (2) how the trends could have changed in each area; (3) if the differences between regions already pointed out still exist.

## 2. Materials and Methods

In order to investigate the paediatricians’ opinions on CF, we designed a specific questionnaire, which was administered through Pediatotem^®^ (Pediatotemweb v. 2.22.0), software developed by Lviiier srl. Pediatotem^®^ is used by almost 1600 PCP all over Italy and is therefore extremely useful for epidemiologic research regarding primary care paediatrics and assisted families. Based on estimates regarding the number of PCP working in Italy during the year 2022 (extracted from national reports from previous years), the total number of active PCP working in Italy during the timespan of the study was considered to be around 7300. Therefore, the Pediatotem^®^ software enabled us to reach almost ¼ of them. The minimum number of respondents was calculated to be 365 PCP in order to achieve a representative sample with a 5% margin of error and 95% confidence interval. The questionnaire was administered during a timespan ranging from January to October 2022 to all the PCP using the Pediatotem software and was filled out on a voluntary basis. The questionnaire was specifically intended to clarify the attitude of PCP towards CF in full-term and healthy babies. It was a slightly modified version of the one designed for the previous inquiry, consisting of four items regarding (1) the method of proposed CF; (2) the suggested age for introduction of CF; (3) the habit of providing parents written information; and (4) the paediatrician’s opinion about the use of baby foods (Table 1).

**Figure 1:**
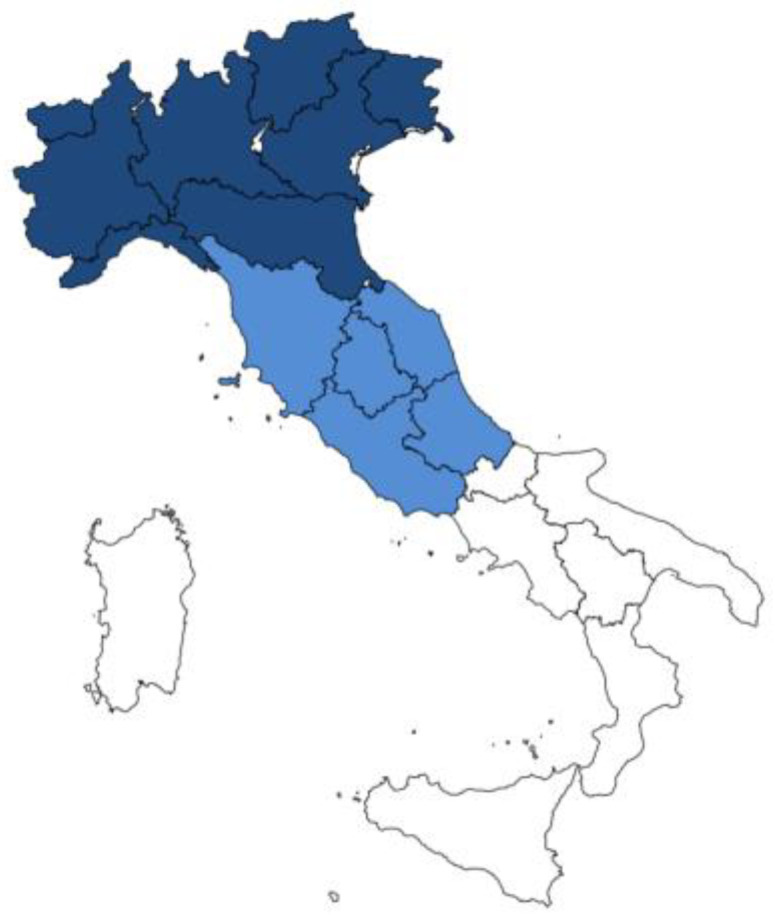
Geographical subdivision of Italy in three areas: North (dark blue), Centre (blue), and South (white).

We divided Italy into three geographical areas based on socio-cultural similarities, as shown in Figure 1.

Categorical variables were tabled, indicating the total number (*n*) and frequency (%). Categorical variables were analysed by 2-way tables, and an χ² test was performed to detect statistically significant differences between the groups. A difference of proportions was performed to compare the results we obtained with the ones from the previous study. Statistical analysis was performed with JASP software (v0.16.2), and *p*-values < 0.01 were considered significant.

## 3. Results

We collected a total of 595/1600 (37%) responses: 225 from Northern, 148 from Central and 222 from Southern Italy. Considering the entirety of PCP working in Italy during the examined timespan, roughly 8% of them filled out the questionnaire (7% of the PCPs from Northern regions, 9% from the Centre and 8.5% from the South of Italy) [9]. The collection of ancillary data, such as the PCP age and the specific district in which they work, was optional, and therefore we were able to get only partial data.

### 3.1. Style of CF

We report an overall rate of 41% (244/595) of paediatricians recommending the traditional spoon-feeding approach, mainly in the South (53.6% of Southern paediatricians). This practice is less common in the North of Italy (25.8%), and Central regions lay in the middle (45.3%). On the other hand, 244 paediatricians (37.6%) suggest spoon-feeding with adult food tastings (49.3% of Northern paediatricians, 32.4% of Central and 29.3% of Southern ones). A considerable share of doctors follows the self-weaning approach (121/595, namely 21.2%), with a prevalence of this habit among the Northern paediatricians (24.9% compared to 22.3% and 16.7% of Central and Southern ones).

### 3.2. Age of CF Introduction

Overall, 544/595 (91.5%) of the interviewed paediatricians suggest starting CF between the 5th and 6th month of life, with a slight predilection for the 5th month (275/595, 46.2%). Only 45 (7.6%) of them propose the introduction of CF at four months of age and six (1%) beyond six months.

### 3.3. Written Information

Of the sample, 512/595 (86.1%) provide families with written information about CF, with no statistical difference between the three areas.

### 3.4. Baby Foods

Some 305/595 (51.3%) of the interviewed paediatricians endorse the use of BFs, mainly in Southern and Central regions.

The results are summarised in Table 2.

### 3.5. Changing Trend

Comparing the results with the ones from the previous study (taking place during the period 2015–2017), we found a nationwide tendency to suggest less traditional feeding and more self-weaning and traditional CF with adult food tastings. The differences showed statistical significance (Table 3). This is also true on a regional basis: the endorsement of traditional spoon-feeding has reduced across the country. On the contrary, the application of self-weaning has increased significantly only in the Centre of the country, and the traditional CF complemented with adult food tastings only in the South; the increase is evident (even if not significant) in the other regions too (Table 4).

The use of baby foods showed a significant reduction, both from a national and regional perspective. The only regions that seem to continue using baby foods as during the previous period are those from Central Italy (Table 3 and Table 4).

Regarding the other items of the questionnaire (the age to start CF and the habit of giving written information to families), the analysis did not point out any significant difference from the previous report.

## 4. Discussion

CF is still a controversial subject in clinical practice. Its importance is widely acknowledged, as it is essential to guarantee adequate growth to children, as well as to favour healthy eating habits in the future, thus preventing several non-communicable diseases in adult life [2]: this becomes even more important considering that in the Italian population, roughly 20% of children between eight and nine years are overweight, almost 10% are obese, and a considerable share do not consume an adequate quantity of fruit and vegetables [10]. Yet, conclusive evidence on the best modality to implement CF is lacking [6]. This can be confusing for families, who can be influenced by the new trends that are gaining popularity (e.g., vegetarian or vegan diets), which may pose a threat to the child’s growth and neurological development if the paediatrician is not involved [11].

From the paediatricians’ perspective, the situation is not much clear either. Even for a widely used method of weaning, such as BLW, there is no agreement on its formal definition. Some authors focus on food consistency (pureed vs. whole pieces) [12], others on the modality of the feeding (parent-led vs baby-led) [13], and some others value the sharing of meals with the rest of the family, which stimulates the child to eat the same food as they do [5,14]. Also, “BLW” is a generic term which encompasses various practices, all sharing the central role of the infant in leading the process of CF. It was first introduced to the international scientific community by Gill Rapley [15], but other modified versions of BLW have been described [16,17], fuelling the uncertainty about what is to be considered BLW and what is not. In this paper, we refer generically to it as the alternative to parent-led weaning (or traditional spoon feeding), mainly intended as its Italian variation, namely “self-weaning” (as illustrated by Lucio Piermarini even before the label of “baby-led weaning” was coined) [17].

The role of paediatricians in supporting the process of CF has already been pointed out [8]. As society evolves, the needs and expectations of parents about this topic change, and recommendations given to families must somehow meet these new demands. Following the growing interest in this topic, we wanted to update the results from our previous study with a new survey sent to PCP. From the data we were able to collect, it emerged that indeed in Italy, the approach of paediatricians to CF has changed over the past few years.

### 4.1. CF Style

Compared to the period 2015–2017, we observed a nationwide reduction in traditional spoon feeding (from 60% to 41%, *p* < 0.01), although it is still the most recommended method across the country. The reduction is more evident in the Northern regions, where it is almost halved (from 48% to 25.8%), where its use is significantly lower compared to the other two regions. On the contrary, there exists a tendency to maintain this habit in the Centre and South of Italy, where it is still by far the most endorsed way of implementing CF.

Conversely, BLW styles became more popular. The increase in self-weaning is more evident in the Central regions (where it has risen from 11% to 22.3%), but also in the North and South, it strengthened its position as an effective and feasible way to wean infants—even if in the South it is still the least chosen method. Now self-weaning appears to be evenly distributed across the country, without significant differences between the regions.

Finally, traditional spoon-feeding with adult food tastings, which represents somehow a middle ground between the other two, obtained a substantial increase (passing from 28% to 37.6%): now it appears to be the most endorsed method in the North of Italy (where it is recommended by almost half of the interviewed paediatricians); also in the other regions the use of this style has increased considerably. This approach seems to be especially appreciated due to the fact that it appropriately balances all macronutrients while at the same time providing a vast arrangement of different foods (and therefore tastes and consistencies). CF with adult food tastings has the advantage of respecting the traditions of every family, which is of capital importance in a multicultural environment as the one modern society is evolving into. Furthermore, knowing that the child might be involved during the family meal may encourage the whole family to adopt a more wholesome diet. In this respect, it is essential for the paediatrician to educate the parents not to add salt to the foods and give their children only foods made with fresh and seasonal ingredients.

Current literature shows little data about how CF is implemented in other countries. For what concerns Europe, a Spanish survey taking place in 2018 highlighted how PCP mostly recommended traditional spoon-feeding and showed a scarce tendency to the routine use of the BLW approach [18]. A more recent study confirmed that in Spain, BLW is not a common choice for weaning: the prevalence of use is low, and more than half of the interviewed mothers had no knowledge of this practice [19]. BLW is also quite uncommon in New Zealand, wherein only between 8% and 18% of parents indicated it as the preferred practice, while the traditional spoon-feeding method was implemented by 70% of parents [13,20]. On the contrary, in the UK, between 30% and 60% of parents strictly follow BLW practices [21,22].

BLW is substantially different from traditional CF, as discussed above. However, the two styles appear to be similar as regards short-term outcomes such as energy intake and risk of choking [4]. It is of capital importance to understand that, based on the currently available scientific evidence, it is not possible to recommend BLW over the traditional approach with the objective of preventing longer-term outcomes such as obesity, or improving children’s growth, as it is not proven to be more effective in achieving these goals either [6,23]. Nevertheless, it is considered to be a safe approach which meets the nutritional needs of the weaning infant, with a few postulated advantages such as lower food fussiness and higher satiety responsiveness. Interestingly, as picky eating during early years has been related to an increased risk of subsequently developing eating disorders such as anorexia nervosa [24], a feeding style which is more baby-led may be of help in reducing this kind of burden. BLW has also proven to be positively associated with language production (eating foods of different textures helps in developing oral and motor skills also required for language production) and comprehension (the BLW approach entails sharing the meal with the rest of the family, which exposes the child to specific interactions and language use) [25] which potentially represents another incentive to resort to this method.

### 4.2. CF Age

The advised age to start weaning has not changed since the 2015–2017 period. Most paediatricians (roughly 90% of the total) still recommend beginning CF between five and six months of age. A few paediatricians recommend an earlier beginning, at four months of age; interestingly enough, they appear to be located mostly in the South (where they represent 12.2%, a significantly higher proportion compared to the other regions).

The suggested age to begin CF is one of the few clear recommendations available about this topic [6,7]. This is directly related to the maturation of both the gastrointestinal tract and the kidney, and, more importantly, to the neurodevelopmental milestones that the child needs to achieve before starting this process safely (the ability to sit unsupported, reach for food, chew, etc.). Consistently with these statements, the age at which CF is generally started has not significantly changed over time in Italian practice. Of note, the most recent recommendations state that CF should be started between 17 and 26 weeks of age (namely between five and six months), but the optimal goal for breastfed infants should be to start at six months of age, considering the adequacy of milk as nourishment until this age and the non-nutritional benefits of mother milk. Human milk is, in fact, rich in compounds that help the immunologic function of the baby (IgA, lactoferrin, HMO), hormones that modulate the metabolic function (such as insulin and leptin), as well as carrying beneficial bacteria contributing to establishing a healthy microbiota, and stem cells [26].

As for the practices carried out in other regions of the world, a survey investigating the habits of physicians towards CF in Middle-East and North Africa showed a high rate of introduction of foods out of the optimal period, namely before four months of age (2%) or after six months (36%) [27]. On the contrary, in Spain, 10.7% of paediatricians suggest starting CF at four months of age, showing similar results to the one obtained in the Southern regions of Italy [18].

From our results, it emerged that Italian paediatricians generally seem to adhere to the WHO’s advice, suggesting beginning CF at six months of age (or during the sixth month of life). There are still a few exceptions, mostly in the South of the country: the explanations could be various, but in our opinion, they could be mainly related to the socio-economic discrepancies between the North and the South. This is bound to the different rates of BLW found in these regions: in previous reports, BLW was shown to be associated with a later introduction of solid foods [28,29]. BLW is also related to a higher rate of breastfeeding [29], and in turn, breastfeeding is (at least in high-income countries) positively associated with the economic condition and educational level of mothers [30]. In fact, from our previous study, it emerged that the breastfeeding rate is lower in the South compared to the North of Italy [8]. In taking all this into account, the current evidence draws attention to the common thread linking the families’ socio-economic condition with BLW, breastfeeding, and compliance with CF recommendations. It is considered that the cited studies focus primarily on the family perspective, while our goal is to assess the conduct of paediatricians; however, it is likely that the recommendations given by doctors are adjusted to the target population they are directed at, supporting the population preferences instead of contrasting them.

### 4.3. CF Written Information

Written information is an easy and convenient way to deliver the mainstays of CF to families. It has a few noteworthy advantages: it is clearer, less open to misinterpretation and always available for parents to consult whenever needed. Nevertheless, it could represent a cause of distance between the paediatrician and the family, as it can be perceived as a manner to standardise a practice that, if anything, every family inevitably must decline into their own personal reality. We attested to a slight reduction in giving written information to families—which nevertheless continues to be the most common habit (about 85% of paediatricians still use this approach nationwide). This could be due to the fact that paediatricians are becoming more aware of the necessity to personalise the practice of CF, in respect of family preferences and multiethnicity, among other things. It is also in line with the increasing endorsement of BLW practices (which are by definition tailored to the child they are addressed to), as stated above.

Among other countries, the practices around this topic are varied. In Spain, paediatricians seem to adopt measures similar to Italy, giving written information to families in 95.3% of cases [18]. On the contrary, in France, the preferred method is to give oral information, with only about 5% of families receiving written information from their paediatrician [31]. This could lead parents to search for advice from other sources, possibly increasing the uncertainty about this topic.

It is fundamental, from the paediatrician’s perspective, to teach families the main nutritional concepts to guarantee an adequate intake of nutrients and healthy eating habits, but it is also of capital importance to empower the parents and encourage them to adopt a responsible approach, which has shown an association with positive outcomes, such as increased satiety responsiveness and lower childhood body mass index (BMI) [23]. Responsible feeding per se does not prevent the development of medical conditions (such as hypertension and type 2 diabetes mellitus) later in life. Even so, it is advisable to promote a responsible approach from the very first months of life and to reinforce it during CF, as it likely contributes to achieving adequate weight during the first years of life [6]. We also believe that a responsible feeding style could help children to become more confident in their approach to food and to foster a bond with their parents.

### 4.4. CF Industrial Baby Foods

The type of food suggested to begin CF is gradually changing. In the period of 2015–2017, we found a tendency to endorse the use of industrial baby foods (roughly two out of every three paediatricians), while now the proportion of paediatricians recommending the use of ready-made products almost equals that of those who do not (51.3% vs. 48.7%, respectively). These differences are more evident if seen from a regional perspective. In the North of Italy, the trend seems to have completely inverted, with 57.8% not recommending commercial baby foods (vs. 40% of the 2015–2017 period). In the South, the paediatricians recommending the use of industrial baby foods are still the majority, but with a significant drop from 81% to 58.1%. For comparison purposes, the survey conducted in the Middle East and North Africa reports 44% of physicians recommending using only homemade foods [27].

Our data seem to confirm the aforementioned trends: in a setting where BLW is becoming more and more popular, paediatricians have to support families by encouraging them to cook healthier foods which can be eaten by infants, thus decreasing the consumption of ready-made baby foods accordingly.

## 5. Conclusions

The most recent data show a general change regarding CF in Italy, at least from the paediatricians’ perspective. BLW—or slight variations of this approach—and traditional spoon feeding with adult food tastings are becoming more and more popular, and this may be due to the increasing evidence supporting some advantages over the traditional weaning method. The use of industrial baby foods varied accordingly over time: in an environment where infants share the foods with the rest of the family, the consumption of ready-made commercial products is supposed to drop, as pointed out by our data. It is unlikely that our study will bring immediate consequences to the current practice regarding CF. Nevertheless, we find that traditional spoon-feeding with adult food tastings shares the benefit of both traditional CF and self-weaning, as it guarantees the appropriate food intake and variety and respects the cultural preferences of families (which represents a key aspect as multiethnicity is becoming one of the most relevant features of our society). We, therefore, deem it the most suitable option for weaning. In future symposiums, we are going to present our results to PCP and our hope is that this “middle way” of weaning will become even more used in the forthcoming years.

Our study has a few limitations. Firstly, the questionnaire was filled out on a voluntary basis, thus creating a selection bias. Secondly, in this study, we did not investigate if the environment in which the paediatricians work (e.g., bigger cities vs smaller towns) is related to different behaviours in CF advice, taking into consideration that weaning habits are influenced by the socio-economic status of the family. Further studies are needed to clarify this point. Third, ancillary data such as PCP’s gender and age were not available for all the participants. Thus, it was not possible to draw conclusions about whether they could influence the habits of PCP or not.

The strong point of this study is that the data come from the real-life practice of the paediatricians interviewed, which permits us to portray an accurate picture of the current state of the art on CF in Italy. The data were collected at a short distance from the last report, giving insight into the changes taking place in this short time span. Moreover, the paediatricians who were included in the study are more evenly distributed around Italy compared to our last report, reducing the biases of the analysis and giving a more accurate depiction of the practice in our country.

From the paediatricians’ perspective, assisting families during CF is a pivotal opportunity to educate them on healthy feeding habits, which could potentially influence long-term health conditions for all family members at all ages. From what is stated above, it appears clear that the habits about CF among Italian paediatricians are radically changing. Interestingly enough, the differences between regions seem to be less evident compared to our previous report. But more importantly, these changes point towards a more responsible approach to CF, consistently with the indications from WHO [32,33] and in line with the latest recommendations published by the main scientific organizations involved. Our hope is that this trend will be maintained in the forthcoming years, with paediatricians being able to support and guide the families in the critical moment of CF and cooperating to ensure that children and their family experience a healthy approach to food. In this regard, the Italian Society of Pediatrics (SIP) and the Italian Society of Pediatric Gastroenterology and Nutrition (SIGENP) promoted cultural campaigns in order to further raise awareness of the importance of CF for the child’s health and well-being.

## Figures and Tables

**Table 1:** Questionnaire sent to Italian primary care paediatricians regarding their habits about CF counselling to families.

**What style of CF do you usually suggest?**			
Traditional spoon-feeding	Traditional with adult food tastings		Self-weaning
**What age do you suggest to begin CF at?**			
4 m (91–120 days)	5 m (121–150 days)	6 m (151–180 days)	>6 m (>180 days)
**Do you provide families with written information about CF?**			
yes		no	
**Do you endorse the use of commercial BFs for CF?**			
yes		no	

**Table 2:** Responses from the PCP who fulfilled the questionnaire during the period January 2022–October 2022.

	Italy		North		Center		Sud		*p*-Value
Total Number	595		225		148		222		
	n	%	n	%	n	%	n	%	
Style									
self-weaning	126	21.2%	56	24.9%	33	22.3%	37	16.7%	0.096
traditional	244	41.0%	58	25.8%	67	45.3%	120	54%	**<0.0001**
traditional w/tastings	224	37.6%	111	49.3%	48	32.4%	65	29.3%	**<0.0001**
Age									
4 months	45	7.6%	12	5.3%	6	4.1%	27	12.2%	**0.004**
5 months	275	46.2%	95	42.2%	70	47.3%	110	49.5%	0.29
6 months	269	45.2%	116	51.6%	68	45.9%	85	38.3%	0.018
>6 months	6	1.0%	2	0.9%	4	2.7%	0	0.0%	0.13
Written information									
yes	512	86.1%	192	85.3%	130	87.8%	190	85.6%	0.77
no	83	13.9%	33	14.7%	18	12.2%	32	14.4%	0.77
Baby foods									
yes	305	51.3%	95	42.2%	81	54.7%	129	58.1%	**0.002**
no	290	48.7%	130	57.8%	67	45.3%	93	41.9%	**0.002**

Statistically significant results are shown in bold.

**Table 3:** Comparison between the results obtained on a national level from the previous (2015–2017) and the current study (January 2022–October 2022).

	Italy				
	2015–2017		2022		*p*-Value
Total Number	665		595		
	n	%	n	%	
Style					
Traditional	397	59.6%	244	41%	**<0.01**
Self-weaning	82	12.3%	126	21.2%	**<0.01**
Traditional with adult food tastings	186	27.9%	244	37.6%	**<0.01**
Age					
4 months	50	7.5%	45	7.6%	0.95
5 months	308	46.3%	275	46.2%	0.94
6 months	297	44.7%	269	45.2%	0.94
>6 months	10	1.5%	6	1%	0.43
Written information					
Yes	604	90.8%	512	86.1%	**0.006**
No	61	9.2%	83	13.9%	**0.006**
Baby foods					
Yes	422	63.5%	305	51.3%	**<0.01**
No	243	36.5%	290	48.7%	**<0.01**

Statistically significant results are shown in bold.

**Table 4:** Comparison between the results obtained in each of the three Italian areas from the previous (2015–2017) and the current study (January 2022–October 2022).

	North					Centre					South				
	2015–2017		2022			2015–2017		2022			2015–2017		2022		
Total Number	207		225			302		148			156		222		
	*n*	%	*n*	%	*p*-Value	*n*	%	*n*	%	*p*-Value	*n*	%	*n*	%	*p*-Value
Style															
Traditional	99	47.8%	58	25.8%	**<0.01**	186	61.6%	67	45.3%	**<0.01**	114	73.1%	119	53.6%	**<0.01**
Self-weaning	33	15.9%	56	24.9%	0.0226	34	11.2%	33	22.3%	**<0.01**	15	9.6%	37	16.7%	0.0644
Traditional w/tastings	75	36.2%	111	49.3%	0.053	82	27.2%	48	32.4%	0.2351	27	17.3%	65	29.3%	**<0.01**
Age															
4 months	7	3.4%	12	5.3%	0.23	21	7%	6	4.1%	0.23	22	14.1%	27	12.2%	0.6
5 months	107	51.6%	95	42.2%	0.04	122	40.4%	70	47.3%	0.2	79	50.6%	110	49.5%	0.77
6 months	91	44%	116	51.6%	0.11	155	51.3%	68	45.9%	0.3	51	32.7%	85	38.3%	0.29
>6 months	2	1%	2	0.9%	0.9	4	1.3%	4	2.7%	0.17	4	2.6%	0	0.0%	0.03
Written information															
Yes	185	89.4%	192	85.3%	0.25	283	93.7%	130	87.8%	0.02	136	87.2%	190	85.6%	0.7
No	22	10.6%	33	14.7%	0.25	19	6.3%	18	12.2%		20	12.8%	32	14.4%	0.7
Baby foods															
Yes	125	60.4%	95	42.2%	**<0.01**	171	56.6%	81	54.7%	0.64	126	80.8%	129	58.1%	**<0.01**
No	82	39.6%	130	57.8%	**<0.01**	131	43.4%	67	45.3%	0.64	30	19.2%	93	41.9%	**<0.01**

Statistically significant differences are shown in bold.

## Data Availability

The data presented in this study are available on request from the corresponding author.
